# [2-(2,2′:4′,2′′-Terpyridin-6′-yl-κ^2^ *N* ^1^,*N* ^1′^)benzoato-κ*O*]manganese(II) trihydrate

**DOI:** 10.1107/S1600536813006004

**Published:** 2013-03-13

**Authors:** Xinzheng Liu

**Affiliations:** a Advanced Material Institute of Research, Department of Chemistry and Chemical Engineering, Qilu Normal University, Shandong 250013, People’s Republic of China

## Abstract

In the title complex, [Mn(C_22_H_14_N_3_O_2_)_2_]·3H_2_O, the Mn^II^ ion is coordinated by two *N*,*N*′,*O*-tridentate 2-(2,2′:4′,2′′-terpyridin-6′-yl-κ^2^ *N* ^1^,*N* ^1′^)benzoate ligands in a distorted *cis*-MnO_2_N_4_ octa­hedral geometry. In one ligand, the dihedral angles between the central pyridine ring, the other bonded pyridine ring, the terminal pyridine ring and the benzene ring are 14.3 (15), 18.3 (18) and 43.9 (16)°, respectively. The equivalent angles in the second ligand are 5.8 (18), 6.3 (18), and 47.0 (17)°, respectively. In the crystal, the complex molecules and lattice water molecules are linked by O—H⋯O and O—H⋯N hydrogen bonds, generating a three-dimensional network.

## Related literature

For background to the applications of coordination complexes, see: Fan *et al.* (2013 ▶).
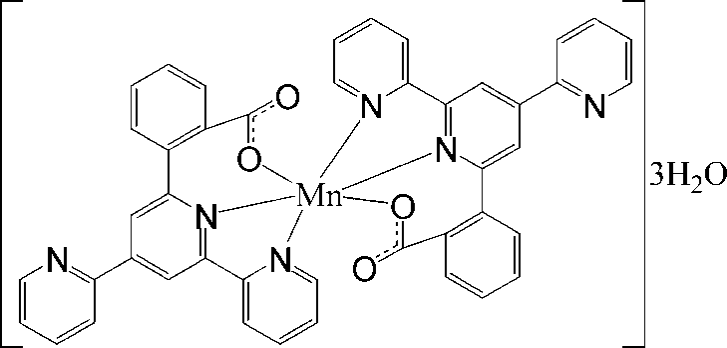


## Experimental

### 

#### Crystal data

[Mn(C_22_H_14_N_3_O_2_)_2_]·3H_2_O
*M* *_r_* = 813.71
Triclinic, 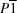

*a* = 11.157 (2) Å
*b* = 12.141 (2) Å
*c* = 15.517 (3) Å
α = 82.79 (3)°
β = 84.19 (3)°
γ = 70.59 (3)°
*V* = 1962.7 (7) Å^3^
*Z* = 2
Mo *K*α radiation
μ = 0.40 mm^−1^
*T* = 293 K
0.12 × 0.10 × 0.08 mm

#### Data collection

Bruker APEXII CCD diffractometer
Absorption correction: multi-scan (*SADABS*; Bruker, 2001 ▶) *T*_min_ = 0.954, *T*_max_ = 0.969
16229 measured reflections
6746 independent reflections
4043 reflections with *I* > 2σ(*I*)
*R*_int_ = 0.098

#### Refinement

*R*[*F* ^2^ > 2σ(*F* ^2^)] = 0.093
*wR*(*F* ^2^) = 0.271
*S* = 1.00
6746 reflections
536 parameters
9 restraints
H atoms treated by a mixture of independent and constrained refinement
Δρ_max_ = 0.58 e Å^−3^
Δρ_min_ = −2.70 e Å^−3^

### 

Data collection: *APEX2* (Bruker, 2004 ▶); cell refinement: *SAINT-Plus* (Bruker, 2001 ▶); data reduction: *SAINT-Plus*; program(s) used to solve structure: *SHELXS97* (Sheldrick, 2008 ▶); program(s) used to refine structure: *SHELXL97* (Sheldrick, 2008 ▶); molecular graphics: *SHELXTL* (Sheldrick, 2008 ▶); software used to prepare material for publication: *SHELXTL*.

## Supplementary Material

Crystal structure: contains datablock(s) global, I. DOI: 10.1107/S1600536813006004/hb7038sup1.cif

Structure factors: contains datablock(s) I. DOI: 10.1107/S1600536813006004/hb7038Isup2.hkl

Additional supplementary materials:  crystallographic information; 3D view; checkCIF report

## Figures and Tables

**Table 1:** Selected bond lengths (Å)

Mn1—O1	2.10 (2)
Mn1—O3	2.11 (2)
Mn1—N5	2.26 (2)
Mn1—N2	2.27 (2)
Mn1—N4	2.33 (2)
Mn1—N1	2.41 (2)

**Table 2:** Hydrogen-bond geometry (Å, °)

*D*—H⋯*A*	*D*—H	H⋯*A*	*D*⋯*A*	*D*—H⋯*A*
O1*W*—H2*W*⋯O2^i^	0.82 (1)	2.23 (8)	2.768 (8)	123 (8)
O1*W*—H1*W*⋯O2^ii^	0.82 (1)	2.27 (7)	2.881 (8)	145 (6)
O2*W*—H4*W*⋯N3	0.82 (1)	2.24 (4)	3.022 (9)	159 (9)
O3*W*—H5*W*⋯O4^iii^	0.82 (1)	2.65 (8)	2.765 (8)	89 (6)

Symmetry codes: (i) 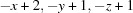; (ii) 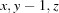; (iii) 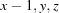.

**Fig. 1.**
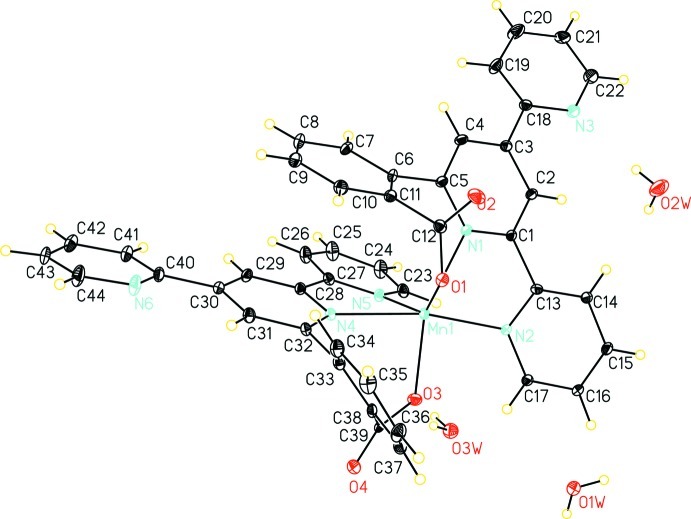
The molecular structure of the title compound with displacement ellipsoids drawn at the 30% probability level.

**Fig. 2.**
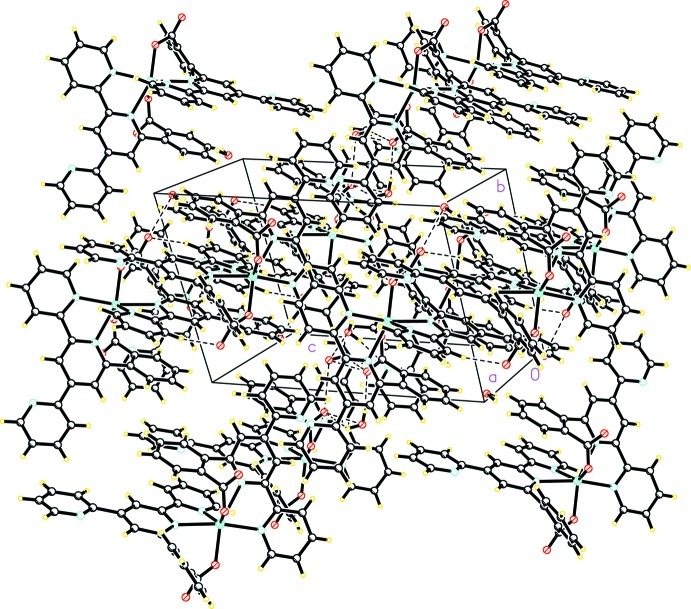
The crystal packing of the title compound, displayed with hydrogen bonds as dashed lines.

## supplementary crystallographic information

### Comment

The design and synthesis of coordination complexes have attracted upsurging research interest not only because of their appealing structural and topological novelty but also owing to their tremendous potential applications in gas storage, microelectronics, ion exchange, chemical separations, nonlinear optics and heterogeneous catalysis (e.g. Fan *et al.*, 2013). Here, we report one new compound: [(C_44_H_28_MnN_6_O_4_).3(H_2_O)], obtained from the solvothermal reaction of 6'-(2-carboxylphenyl)-2,2':4',2''-terpyridine and manganese(II) sulfate.

The title compound, [(C_44_H_28_MnN_6_O_4_).3(H_2_O)], consists of one Mn(II), two 6'-(2-carboxylphenyl)-2,2':4',2''-terpyridine, and three uncoordinated water molecules. Mn(1) owns a distorted octahedral coordination geometry, completed by four N atoms and two O atoms from two depornated 6'-(2-carboxylphenyl)-2,2':4',2''-terpyridine (Figure 1). The Mn—O and Mn—N distances are in the range of 2.10 (2)–2.11 (2) and 2.26 (2)–2.41 (2) Å, respectively. O—H···O and O—H···N hydrogen bonding in the packing diagram leads to a consolidation of the structure (Fig. 2; Table 2) .

### Experimental

A mixture of 6'-(2-carboxylphenyl)-2,2':4',2''-terpyridine (0.10 mmol, 0.035 g), manganese(II) sulfate monohydrate (0.10 mmol, 0.017 g), NaOH (0.20 mmol, 0.008 g) and 12 ml H_2_O was placed in a Teflon-lined stainless steel vessel, heated to 170°C for 3 days, followed by slow cooling (a descent rate of 10°C/h) to room temperature to yield orange blocks. Anal. Calc. for C_44_H_34_MnN_6_O_7_: C 64.95, H 4.21, N 10.33%; Found: C 64.91, H 4.16, N 10.28%.

### Refinement

All hydrogen atoms bound to carbon were refined using a riding model with distance C—H = 0.93 Å, *U*_iso_ = 1.2*U*_eq_ (C) for aromatic atoms. The H atoms of the water molecule were located from difference density maps and were refined with d(O—H) = 0.83 (2) Å, and with a fixed *U*_iso_ of 0.80 Å^2^.

### Crystal data

**Table:**

[Mn(C_22_H_14_N_3_O_2_)_2_]·3H_2_O	*Z* = 2
*M**_r_* = 813.71	*F*(000) = 842
Triclinic, *P*1	*D*_x_ = 1.377 Mg m^−^^3^
Hall symbol: -P 1	Mo *K*α radiation, λ = 0.71073 Å
*a* = 11.157 (2) Å	Cell parameters from 6746 reflections
*b* = 12.141 (2) Å	θ = 3.0–25.0°
*c* = 15.517 (3) Å	µ = 0.40 mm^−^^1^
α = 82.79 (3)°	*T* = 293 K
β = 84.19 (3)°	Block, orange
γ = 70.59 (3)°	0.12 × 0.10 × 0.08 mm
*V* = 1962.7 (7) Å^3^	

### Data collection

**Table:**

Bruker APEXII CCD diffractometer	6746 independent reflections
Radiation source: fine-focus sealed tube	4043 reflections with *I* > 2σ(*I*)
Graphite monochromator	*R*_int_ = 0.098
φ and ω scans	θ_max_ = 25.0°, θ_min_ = 3.0°
Absorption correction: multi-scan (*SADABS*; Bruker, 2001)	*h* = −13→13
*T*_min_ = 0.954, *T*_max_ = 0.969	*k* = −14→14
16229 measured reflections	*l* = −18→18

### Refinement

**Table:**

Refinement on *F*^2^	Primary atom site location: structure-invariant direct methods
Least-squares matrix: full	Secondary atom site location: difference Fourier map
*R*[*F*^2^ > 2σ(*F*^2^)] = 0.093	Hydrogen site location: inferred from neighbouring sites
*wR*(*F*^2^) = 0.271	H atoms treated by a mixture of independent and constrained refinement
*S* = 1.00	*w* = 1/[σ^2^(*F*_o_^2^) + (0.139*P*)^2^ + 1.6146*P*] where *P* = (*F*_o_^2^ + 2*F*_c_^2^)/3
6746 reflections	(Δ/σ)_max_ = 0.001
536 parameters	Δρ_max_ = 0.58 e Å^−^^3^
9 restraints	Δρ_min_ = −2.70 e Å^−^^3^

### Special details

**Table:**

Geometry. All esds (except the esd in the dihedral angle between two l.s. planes) are estimated using the full covariance matrix. The cell esds are taken into account individually in the estimation of esds in distances, angles and torsion angles; correlations between esds in cell parameters are only used when they are defined by crystal symmetry. An approximate (isotropic) treatment of cell esds is used for estimating esds involving l.s. planes.
Refinement. Refinement of F^2^ against ALL reflections. The weighted R-factor wR and goodness of fit S are based on F^2^, conventional R-factors R are based on F, with F set to zero for negative F^2^. The threshold expression of F^2^ > 2sigma(F^2^) is used only for calculating R-factors(gt) etc. and is not relevant to the choice of reflections for refinement. R-factors based on F^2^ are statistically about twice as large as those based on F, and R- factors based on ALL data will be even larger.

### Fractional atomic coordinates and isotropic or equivalent isotropic displacement parameters (Å^2^)

**Table:**

	*x*	*y*	*z*	*U*_iso_*/*U*_eq_	
C1	0.728 (3)	0.730 (3)	0.6259 (19)	0.033 (7)	
C2	0.605 (3)	0.805 (3)	0.608 (2)	0.042 (8)	
H2	0.5575	0.7851	0.5705	0.050*	
C3	0.556 (3)	0.910 (3)	0.646 (2)	0.039 (7)	
C4	0.634 (3)	0.935 (3)	0.700 (2)	0.041 (8)	
H4	0.6056	1.0058	0.7249	0.049*	
C5	0.753 (3)	0.858 (3)	0.715 (2)	0.036 (7)	
C6	0.840 (3)	0.885 (3)	0.7698 (19)	0.039 (7)	
C7	0.792 (4)	0.934 (3)	0.850 (2)	0.052 (9)	
H7	0.7068	0.9482	0.8678	0.063*	
C8	0.870 (4)	0.962 (3)	0.900 (2)	0.061 (11)	
H8	0.8378	0.9925	0.9530	0.074*	
C9	0.996 (5)	0.943 (3)	0.873 (3)	0.067 (12)	
H9	1.0486	0.9625	0.9074	0.081*	
C10	1.046 (3)	0.896 (3)	0.795 (2)	0.051 (9)	
H10	1.1310	0.8852	0.7773	0.061*	
C11	0.970 (3)	0.865 (3)	0.743 (2)	0.036 (7)	
C12	1.027 (3)	0.814 (3)	0.659 (2)	0.038 (7)	
C13	0.784 (3)	0.615 (3)	0.588 (2)	0.034 (7)	
C14	0.732 (3)	0.587 (3)	0.520 (2)	0.042 (8)	
H14	0.6611	0.6409	0.4952	0.051*	
C15	0.788 (3)	0.477 (3)	0.490 (2)	0.048 (9)	
H15	0.7549	0.4570	0.4442	0.058*	
C16	0.893 (3)	0.400 (3)	0.528 (2)	0.043 (8)	
H16	0.9304	0.3257	0.5088	0.052*	
C17	0.941 (3)	0.433 (3)	0.596 (2)	0.036 (7)	
H17	1.0134	0.3798	0.6209	0.043*	
C18	0.422 (3)	0.987 (3)	0.631 (2)	0.042 (8)	
C19	0.357 (4)	1.069 (4)	0.685 (3)	0.070 (13)	
H19	0.3948	1.0809	0.7320	0.084*	
C20	0.232 (4)	1.137 (4)	0.667 (3)	0.082 (15)	
H20	0.1865	1.1956	0.7028	0.098*	
C21	0.176 (3)	1.119 (3)	0.601 (3)	0.062 (11)	
H21	0.0916	1.1618	0.5901	0.075*	
C22	0.248 (3)	1.035 (4)	0.548 (3)	0.061 (10)	
H22	0.2125	1.0238	0.4998	0.073*	
C23	0.724 (3)	0.575 (3)	0.834 (2)	0.045 (8)	
H23	0.7145	0.5443	0.7836	0.054*	
C24	0.625 (3)	0.600 (4)	0.894 (2)	0.061 (11)	
H24	0.5496	0.5881	0.8860	0.074*	
C25	0.639 (3)	0.644 (5)	0.969 (3)	0.067 (13)	
H25	0.5725	0.6626	1.0112	0.080*	
C26	0.754 (3)	0.660 (4)	0.980 (2)	0.055 (10)	
H26	0.7636	0.6922	1.0295	0.066*	
C27	0.853 (3)	0.629 (3)	0.9173 (19)	0.037 (7)	
C28	0.982 (3)	0.637 (3)	0.9267 (18)	0.033 (7)	
C29	1.014 (3)	0.670 (3)	1.0000 (19)	0.039 (7)	
H29	0.9530	0.6923	1.0458	0.046*	
C30	1.139 (3)	0.671 (3)	1.0061 (19)	0.038 (7)	
C31	1.225 (3)	0.636 (3)	0.937 (2)	0.041 (8)	
H31	1.3082	0.6350	0.9382	0.049*	
C32	1.189 (3)	0.600 (3)	0.8639 (18)	0.034 (7)	
C33	1.285 (3)	0.557 (3)	0.7919 (19)	0.037 (7)	
C34	1.369 (3)	0.617 (4)	0.761 (2)	0.050 (9)	
H34	1.3612	0.6865	0.7831	0.060*	
C35	1.464 (4)	0.576 (4)	0.696 (3)	0.065 (12)	
H35	1.5175	0.6189	0.6742	0.078*	
C36	1.479 (3)	0.472 (4)	0.664 (2)	0.059 (10)	
H36	1.5450	0.4435	0.6226	0.070*	
C37	1.397 (3)	0.409 (4)	0.693 (2)	0.054 (9)	
H37	1.4077	0.3381	0.6719	0.064*	
C38	1.298 (3)	0.453 (3)	0.7553 (19)	0.036 (7)	
C39	1.210 (3)	0.382 (3)	0.7794 (18)	0.036 (7)	
C40	1.180 (3)	0.706 (3)	1.083 (2)	0.043 (8)	
C41	1.101 (4)	0.729 (3)	1.160 (2)	0.047 (8)	
H41	1.0187	0.7253	1.1625	0.057*	
C42	1.145 (4)	0.759 (4)	1.230 (2)	0.058 (10)	
H42	1.0929	0.7774	1.2806	0.069*	
C43	1.269 (4)	0.759 (4)	1.225 (2)	0.061 (11)	
H43	1.3030	0.7746	1.2726	0.073*	
C44	1.339 (4)	0.736 (5)	1.149 (3)	0.074 (13)	
H44	1.4221	0.7383	1.1466	0.089*	
Mn1	0.9818 (4)	0.5939 (4)	0.7297 (3)	0.0315 (15)	
N1	0.800 (2)	0.754 (2)	0.6797 (15)	0.033 (6)	
N2	0.888 (2)	0.539 (2)	0.6268 (15)	0.034 (6)	
N3	0.370 (3)	0.968 (3)	0.564 (2)	0.052 (7)	
N4	1.068 (2)	0.604 (2)	0.8581 (15)	0.031 (5)	
N5	0.837 (2)	0.591 (2)	0.8421 (16)	0.036 (6)	
N6	1.299 (3)	0.711 (3)	1.079 (2)	0.063 (9)	
O1	1.0765 (19)	0.7039 (19)	0.6607 (13)	0.038 (5)	
O2	1.023 (3)	0.885 (2)	0.5930 (17)	0.066 (7)	
O3	1.104 (2)	0.4190 (19)	0.7432 (15)	0.045 (5)	
O4	1.244 (3)	0.289 (2)	0.8273 (15)	0.058 (7)	
O1W	0.867 (3)	0.128 (2)	0.5750 (18)	0.063 (7)	
O2W	0.409 (4)	0.792 (4)	0.433 (3)	0.106 (13)	
H3W	0.40 (3)	0.734 (17)	0.46 (2)	0.080*	
H4W	0.42 (5)	0.84 (3)	0.464 (19)	0.080*	
H2W	0.84 (4)	0.15 (2)	0.527 (12)	0.080*	
H1W	0.88 (4)	0.060 (10)	0.59 (2)	0.080*	
O3W	0.438 (3)	0.099 (3)	0.894 (2)	0.080*	
H5W	0.367 (19)	0.11 (4)	0.94 (2)	0.2 (3)*	
H6W	0.50 (4)	0.13 (2)	0.91 (3)	0.080*	

### Atomic displacement parameters (Å^2^)

**Table:**

	*U*^11^	*U*^22^	*U*^33^	*U*^12^	*U*^13^	*U*^23^
C1	0.031 (15)	0.033 (16)	0.033 (16)	−0.005 (13)	−0.007 (12)	−0.009 (12)
C2	0.041 (18)	0.036 (18)	0.046 (19)	−0.003 (15)	−0.013 (15)	−0.014 (14)
C3	0.034 (16)	0.037 (18)	0.042 (18)	−0.001 (14)	−0.006 (14)	−0.008 (14)
C4	0.035 (16)	0.031 (17)	0.05 (2)	−0.001 (14)	−0.006 (14)	−0.016 (14)
C5	0.031 (15)	0.033 (17)	0.042 (18)	−0.006 (13)	−0.004 (13)	−0.012 (13)
C6	0.047 (18)	0.042 (19)	0.030 (16)	−0.014 (16)	−0.009 (14)	−0.012 (13)
C7	0.06 (2)	0.05 (2)	0.05 (2)	−0.003 (18)	−0.007 (17)	−0.024 (16)
C8	0.08 (3)	0.06 (2)	0.04 (2)	−0.01 (2)	−0.018 (19)	−0.022 (17)
C9	0.08 (3)	0.05 (2)	0.07 (3)	−0.01 (2)	−0.04 (2)	−0.02 (2)
C10	0.048 (19)	0.05 (2)	0.06 (2)	−0.019 (17)	−0.024 (17)	−0.007 (17)
C11	0.037 (16)	0.033 (17)	0.044 (18)	−0.015 (14)	−0.008 (13)	−0.005 (13)
C12	0.037 (17)	0.041 (19)	0.034 (17)	−0.011 (15)	0.001 (13)	0.000 (14)
C13	0.030 (15)	0.029 (16)	0.039 (17)	−0.004 (13)	−0.002 (13)	−0.004 (13)
C14	0.044 (18)	0.037 (18)	0.043 (18)	−0.003 (15)	−0.015 (14)	−0.010 (14)
C15	0.047 (19)	0.05 (2)	0.044 (19)	−0.003 (17)	−0.013 (15)	−0.021 (16)
C16	0.06 (2)	0.033 (17)	0.037 (18)	−0.006 (16)	−0.005 (15)	−0.012 (13)
C17	0.039 (16)	0.031 (16)	0.039 (17)	−0.010 (14)	−0.009 (13)	−0.009 (13)
C18	0.040 (17)	0.032 (17)	0.06 (2)	−0.008 (15)	−0.016 (15)	−0.007 (14)
C19	0.05 (2)	0.08 (3)	0.07 (3)	0.01 (2)	−0.018 (19)	−0.05 (2)
C20	0.05 (2)	0.08 (3)	0.11 (4)	0.01 (2)	−0.02 (2)	−0.06 (3)
C21	0.037 (19)	0.05 (2)	0.09 (3)	0.004 (18)	−0.03 (2)	−0.02 (2)
C22	0.042 (19)	0.06 (3)	0.07 (3)	−0.005 (19)	−0.018 (18)	−0.02 (2)
C23	0.034 (17)	0.06 (2)	0.05 (2)	−0.014 (17)	−0.013 (14)	−0.004 (16)
C24	0.031 (17)	0.11 (3)	0.05 (2)	−0.03 (2)	0.003 (16)	−0.01 (2)
C25	0.031 (18)	0.12 (4)	0.05 (2)	−0.02 (2)	0.004 (16)	−0.01 (2)
C26	0.042 (19)	0.09 (3)	0.035 (19)	−0.02 (2)	−0.002 (15)	−0.007 (18)
C27	0.031 (16)	0.045 (19)	0.030 (16)	−0.009 (14)	−0.002 (12)	0.001 (13)
C28	0.034 (15)	0.038 (17)	0.028 (15)	−0.011 (14)	−0.001 (12)	−0.004 (12)
C29	0.039 (17)	0.046 (19)	0.031 (16)	−0.012 (15)	0.003 (13)	−0.014 (14)
C30	0.044 (18)	0.037 (18)	0.031 (16)	−0.010 (15)	−0.006 (13)	−0.008 (13)
C31	0.035 (16)	0.05 (2)	0.041 (18)	−0.020 (16)	−0.007 (14)	−0.006 (15)
C32	0.036 (16)	0.041 (18)	0.026 (15)	−0.012 (14)	−0.006 (12)	0.000 (12)
C33	0.027 (14)	0.05 (2)	0.033 (16)	−0.010 (14)	−0.004 (12)	−0.002 (14)
C34	0.048 (19)	0.07 (2)	0.04 (2)	−0.025 (19)	−0.002 (16)	−0.003 (17)
C35	0.05 (2)	0.10 (4)	0.06 (2)	−0.04 (2)	0.001 (18)	0.00 (2)
C36	0.04 (2)	0.09 (3)	0.05 (2)	−0.02 (2)	0.007 (16)	−0.03 (2)
C37	0.043 (19)	0.07 (3)	0.04 (2)	−0.001 (19)	−0.010 (15)	−0.014 (17)
C38	0.027 (14)	0.052 (19)	0.026 (15)	−0.007 (14)	−0.005 (12)	−0.010 (13)
C39	0.043 (17)	0.035 (17)	0.023 (15)	0.001 (14)	−0.003 (13)	−0.012 (13)
C40	0.05 (2)	0.05 (2)	0.032 (17)	−0.021 (17)	−0.011 (15)	−0.007 (14)
C41	0.06 (2)	0.06 (2)	0.033 (18)	−0.029 (19)	0.001 (15)	−0.013 (15)
C42	0.08 (3)	0.07 (3)	0.04 (2)	−0.04 (2)	0.005 (18)	−0.016 (17)
C43	0.08 (3)	0.07 (3)	0.04 (2)	−0.02 (2)	−0.021 (19)	−0.021 (18)
C44	0.06 (2)	0.11 (4)	0.07 (3)	−0.03 (3)	−0.01 (2)	−0.04 (3)
Mn1	0.032 (2)	0.035 (3)	0.027 (2)	−0.009 (2)	−0.0035 (17)	−0.0057 (18)
N1	0.033 (13)	0.031 (14)	0.034 (14)	−0.009 (11)	−0.003 (10)	−0.007 (10)
N2	0.041 (14)	0.029 (13)	0.031 (13)	−0.007 (11)	−0.005 (11)	−0.010 (10)
N3	0.042 (16)	0.051 (18)	0.060 (19)	−0.004 (14)	−0.017 (14)	−0.014 (14)
N4	0.031 (13)	0.036 (14)	0.028 (13)	−0.013 (11)	−0.003 (10)	−0.001 (10)
N5	0.032 (13)	0.043 (15)	0.035 (14)	−0.013 (12)	−0.006 (10)	−0.007 (11)
N6	0.053 (18)	0.10 (3)	0.050 (19)	−0.035 (19)	−0.006 (14)	−0.029 (18)
O1	0.035 (11)	0.043 (14)	0.039 (12)	−0.015 (11)	−0.001 (9)	−0.007 (9)
O2	0.082 (19)	0.052 (16)	0.048 (16)	−0.010 (14)	0.010 (13)	0.014 (12)
O3	0.037 (12)	0.038 (13)	0.056 (14)	−0.006 (10)	−0.007 (10)	−0.008 (10)
O4	0.078 (18)	0.046 (15)	0.043 (14)	−0.011 (14)	−0.013 (13)	0.001 (11)
O1W	0.076 (19)	0.057 (17)	0.056 (17)	−0.015 (16)	−0.012 (14)	−0.011 (13)
O2W	0.10 (3)	0.09 (3)	0.11 (3)	0.00 (2)	0.01 (2)	−0.05 (2)

### Geometric parameters (Å, º)

**Table:**

C1—N1	1.34 (4)	C25—C26	1.39 (5)
C1—C2	1.41 (4)	C25—H25	0.9300
C1—C13	1.49 (4)	C26—C27	1.39 (5)
C2—C3	1.39 (4)	C26—H26	0.9300
C2—H2	0.9300	C27—N5	1.35 (4)
C3—C4	1.39 (4)	C27—C28	1.50 (4)
C3—C18	1.50 (4)	C28—N4	1.36 (4)
C4—C5	1.38 (4)	C28—C29	1.37 (4)
C4—H4	0.9300	C29—C30	1.42 (4)
C5—N1	1.35 (4)	C29—H29	0.9300
C5—C6	1.49 (4)	C30—C31	1.38 (4)
C6—C11	1.41 (4)	C30—C40	1.48 (4)
C6—C7	1.42 (4)	C31—C32	1.40 (4)
C7—C8	1.37 (5)	C31—H31	0.9300
C7—H7	0.9300	C32—N4	1.35 (4)
C8—C9	1.38 (6)	C32—C33	1.49 (4)
C8—H8	0.9300	C33—C34	1.39 (5)
C9—C10	1.39 (6)	C33—C38	1.40 (4)
C9—H9	0.9300	C34—C35	1.40 (5)
C10—C11	1.40 (4)	C34—H34	0.9300
C10—H10	0.9300	C35—C36	1.36 (6)
C11—C12	1.50 (4)	C35—H35	0.9300
C12—O2	1.24 (4)	C36—C37	1.39 (6)
C12—O1	1.27 (4)	C36—H36	0.9300
C13—N2	1.35 (4)	C37—C38	1.39 (5)
C13—C14	1.38 (4)	C37—H37	0.9300
C14—C15	1.39 (4)	C38—C39	1.50 (5)
C14—H14	0.9300	C39—O4	1.24 (4)
C15—C16	1.36 (5)	C39—O3	1.28 (4)
C15—H15	0.9300	C40—N6	1.34 (4)
C16—C17	1.39 (4)	C40—C41	1.40 (5)
C16—H16	0.9300	C41—C42	1.38 (5)
C17—N2	1.36 (4)	C41—H41	0.9300
C17—H17	0.9300	C42—C43	1.37 (6)
C18—N3	1.33 (4)	C42—H42	0.9300
C18—C19	1.35 (5)	C43—C44	1.35 (6)
C19—C20	1.40 (5)	C43—H43	0.9300
C19—H19	0.9300	C44—N6	1.33 (5)
C20—C21	1.33 (6)	C44—H44	0.9300
C20—H20	0.9300	Mn1—O1	2.10 (2)
C21—C22	1.37 (5)	Mn1—O3	2.11 (2)
C21—H21	0.9300	Mn1—N5	2.26 (2)
C22—N3	1.36 (4)	Mn1—N2	2.27 (2)
C22—H22	0.9300	Mn1—N4	2.33 (2)
C23—N5	1.36 (4)	Mn1—N1	2.41 (2)
C23—C24	1.36 (5)	O1W—H2W	0.819 (18)
C23—H23	0.9300	O1W—H1W	0.82 (2)
C24—C25	1.37 (5)	O2W—H3W	0.821 (17)
C24—H24	0.9300	O2W—H4W	0.82 (2)
			
N1—C1—C2	122 (3)	C29—C28—C27	123 (3)
N1—C1—C13	116 (2)	C28—C29—C30	120 (3)
C2—C1—C13	122 (3)	C28—C29—H29	120.0
C1—C2—C3	120 (3)	C30—C29—H29	120.0
C1—C2—H2	120.3	C31—C30—C29	117 (3)
C3—C2—H2	120.2	C31—C30—C40	120 (3)
C4—C3—C2	117 (3)	C29—C30—C40	123 (3)
C4—C3—C18	123 (3)	C30—C31—C32	121 (3)
C2—C3—C18	120 (3)	C30—C31—H31	119.4
C3—C4—C5	121 (3)	C32—C31—H31	119.6
C3—C4—H4	119.6	N4—C32—C31	121 (3)
C5—C4—H4	119.5	N4—C32—C33	119 (2)
N1—C5—C4	122 (3)	C31—C32—C33	120 (3)
N1—C5—C6	116 (3)	C34—C33—C38	118 (3)
C4—C5—C6	122 (3)	C34—C33—C32	120 (3)
C11—C6—C7	119 (3)	C38—C33—C32	123 (3)
C11—C6—C5	121 (3)	C33—C34—C35	121 (4)
C7—C6—C5	120 (3)	C33—C34—H34	119.2
C8—C7—C6	121 (4)	C35—C34—H34	119.3
C8—C7—H7	119.7	C36—C35—C34	120 (4)
C6—C7—H7	119.6	C36—C35—H35	120.0
C9—C8—C7	120 (4)	C34—C35—H35	120.0
C9—C8—H8	120.2	C35—C36—C37	120 (3)
C7—C8—H8	119.9	C35—C36—H36	119.8
C8—C9—C10	121 (3)	C37—C36—H36	119.9
C8—C9—H9	119.7	C36—C37—C38	120 (4)
C10—C9—H9	119.5	C36—C37—H37	120.1
C9—C10—C11	121 (4)	C38—C37—H37	120.2
C9—C10—H10	119.9	C33—C38—C37	121 (3)
C11—C10—H10	119.5	C33—C38—C39	123 (3)
C6—C11—C10	119 (3)	C37—C38—C39	116 (3)
C6—C11—C12	122 (3)	O4—C39—O3	123 (3)
C10—C11—C12	119 (3)	O4—C39—C38	121 (3)
O2—C12—O1	125 (3)	O3—C39—C38	116 (3)
O2—C12—C11	117 (3)	N6—C40—C41	121 (3)
O1—C12—C11	118 (3)	N6—C40—C30	117 (3)
N2—C13—C14	122 (3)	C41—C40—C30	122 (3)
N2—C13—C1	115 (3)	C42—C41—C40	120 (3)
C14—C13—C1	122 (3)	C42—C41—H41	120.3
C13—C14—C15	119 (3)	C40—C41—H41	120.1
C13—C14—H14	120.5	C43—C42—C41	119 (3)
C15—C14—H14	120.5	C43—C42—H42	120.7
C16—C15—C14	120 (3)	C41—C42—H42	120.7
C16—C15—H15	120.3	C42—C43—C44	118 (3)
C14—C15—H15	120.2	C42—C43—H43	120.6
C15—C16—C17	119 (3)	C44—C43—H43	121.0
C15—C16—H16	120.4	N6—C44—C43	125 (4)
C17—C16—H16	120.4	N6—C44—H44	117.5
N2—C17—C16	122 (3)	C43—C44—H44	117.4
N2—C17—H17	118.9	O1—Mn1—O3	110.7 (9)
C16—C17—H17	118.9	O1—Mn1—N5	141.2 (9)
N3—C18—C19	122 (3)	O3—Mn1—N5	100.9 (9)
N3—C18—C3	116 (3)	O1—Mn1—N2	104.9 (9)
C19—C18—C3	122 (3)	O3—Mn1—N2	87.1 (9)
C18—C19—C20	119 (4)	N5—Mn1—N2	98.5 (9)
C18—C19—H19	120.6	O1—Mn1—N4	90.8 (8)
C20—C19—H19	120.7	O3—Mn1—N4	82.7 (9)
C21—C20—C19	121 (4)	N5—Mn1—N4	71.0 (8)
C21—C20—H20	119.5	N2—Mn1—N4	163.5 (9)
C19—C20—H20	119.5	O1—Mn1—N1	80.6 (8)
C22—C21—C20	117 (3)	O3—Mn1—N1	156.2 (9)
C22—C21—H21	121.3	N5—Mn1—N1	79.2 (9)
C20—C21—H21	121.6	N2—Mn1—N1	69.5 (8)
C21—C22—N3	123 (4)	N4—Mn1—N1	119.0 (8)
C21—C22—H22	118.4	C1—N1—C5	118 (2)
N3—C22—H22	118.5	C1—N1—Mn1	116.3 (18)
N5—C23—C24	123 (3)	C5—N1—Mn1	124.1 (19)
N5—C23—H23	118.3	C17—N2—C13	118 (2)
C24—C23—H23	118.4	C17—N2—Mn1	120.5 (19)
C25—C24—C23	119 (3)	C13—N2—Mn1	121.4 (19)
C25—C24—H24	120.6	C18—N3—C22	118 (3)
C23—C24—H24	120.5	C32—N4—C28	119 (2)
C24—C25—C26	119 (3)	C32—N4—Mn1	124.5 (18)
C24—C25—H25	120.3	C28—N4—Mn1	115.6 (18)
C26—C25—H25	120.2	C23—N5—C27	118 (3)
C25—C26—C27	119 (3)	C23—N5—Mn1	123 (2)
C25—C26—H26	120.4	C27—N5—Mn1	117.7 (19)
C27—C26—H26	120.4	C40—N6—C44	118 (3)
N5—C27—C26	121 (3)	C12—O1—Mn1	120.0 (18)
N5—C27—C28	116 (2)	C39—O3—Mn1	126 (2)
C26—C27—C28	123 (3)	H2W—O1W—H1W	114.5 (16)
N4—C28—C29	122 (3)	H3W—O2W—H4W	115 (3)
N4—C28—C27	115 (2)		

### Hydrogen-bond geometry (Å, º)

**Table:**

*D*—H···*A*	*D*—H	H···*A*	*D*···*A*	*D*—H···*A*
O1*W*—H2*W*···O2^i^	0.82 (1)	2.23 (8)	2.768 (8)	123 (8)
O1*W*—H1*W*···O2^ii^	0.82 (1)	2.27 (7)	2.881 (8)	145 (6)
O2*W*—H4*W*···N3	0.82 (1)	2.24 (4)	3.022 (9)	159 (9)
O3*W*—H5*W*···O4^iii^	0.82 (1)	2.65 (8)	2.765 (8)	89 (6)

Symmetry codes: (i) −*x*+2, −*y*+1, −*z*+1; (ii) *x*, *y*−1, *z*; (iii) *x*−1, *y*, *z*.
